# Delirium diagnosis and handover to primary care providers and medical teams

**DOI:** 10.1192/bjo.2021.137

**Published:** 2021-06-18

**Authors:** Saba Inam, Joanne Flood, Aine Butler

**Affiliations:** 1 Highfield Healthcare, Whitehall, North Dublin Mental Health Services for Older Persons, Willow day hospital, Ashlin Centre, Beaumont Road; 2 North Dublin Mental Health Services for Older Persons, Willow day hospital, Ashlin Centre, Beaumont Road

## Abstract

**Aims:**

Delirium is a common medical problem with a prevalence of over 50% in over 65s admitted to general hospitals (1,2) . Delirium is linked with poor clinical outcome, including increased risk of falls, prolonged admissions and an overall increased risk of morbidity and mortality (2,3,4). Delirium in older adults is also associated with an increased rate of cognitive decline, future risk of cognitive decline and a risk of depression (5,6,7). There is potential to improve clinical practice by improving assessment and management of delirium. It is imperative that where delirium is detected, it should be clearly documented to aid handover to primary care providers and medical teams (8,9).

**Method:**

The standard for this audit was set according to SIGN 157 (9). Data were collected retrospectively from consults sent to a liaison psychiatry of old age service within an acute hospital setting. The medical discharge summaries from July to December 2019 were reviewed. Two key data points were collated, the diagnoses of delirium by either medical or liaison psychiatry team and the inclusion of this diagnosis in the patient discharge summaries. An updated delirium protocol was devised and introduced in the hospital setting in January 2020 to include tools for effective diagnosis of delirium and instruction to include this diagnosis if made in patient's discharge summaries. Re-audit was initiated following the introduction of the updated delirium protocol for the period of January to March 2020.

**Result:**

A total of 116 patients were assessed from July to December 2019. 102 discharge summaries were available for review for the purpose of this audit. Prior to the introduction of the updated delirium protocol, delirium was diagnosed by the liaison team in 57% of all referrals. Delirium was underdiagnosed by medical teams in 73% of those subsequently diagnosed. The diagnosis of delirium was present in 42% of all discharge summaries to primary care providers. Subsequent to the introduction of the updated protocol, delirium was diagnosed in 48% of all liaison referrals during the time period specified. The proportion of under-diagnosis of delirium by medical teams stayed at 73%, the diagnosis of delirium was present in 53% of discharge summaries.

**Conclusion:**

The recognition and diagnosis of delirium in the general medical setting continues to be a key issue in the management of older adults. The importance of this diagnosis and it's associated after effects needs to be disseminated amongst all care providers. Greater efforts to enhance these aspects of delirium management in the acute hospital setting are required.

